# Systemic benefit of radiation therapy *via* abscopal effect

**DOI:** 10.3389/fonc.2022.987142

**Published:** 2022-10-25

**Authors:** Daniel J. Craig, Stephanie Ambrose, Laura Stanbery, Adam Walter, John Nemunaitis

**Affiliations:** ^1^ University of Toledo, Department of Internal Medicine, Toledo, OH, United States; ^2^ Medical Affairs, Gradalis, Inc., Carrollton, TX, United States; ^3^ Gynecologic Oncology, Promedica, Toledo, OH, United States

**Keywords:** abscopal effect, irradiation, cancer management, immune response, autologous tumor immunotherapy, radiation therapy

## Abstract

Evidence of a systemic response related to localized radiation therapy (RT) in cancer management is rare. However, enhancing the immune response *via* immunotherapy followed by localized RT has shown evidence of tumor shrinkage to non-irradiated metastatic disease thereby inducing an “abscopal effect.” Combined induction of the cGAS-STING pathway and activation of IFN-gamma signaling cascade related to RT within an activated immune environment promotes neoantigen presentation and expansion of cytotoxic effector cells enabling enhancement of systemic immune response. A proposed mechanism, case examples, and clinical trial evidence of “abscopal effect” benefit are reviewed. Results support strategic therapeutic testing to enhance “abscopal effect.”

## Introduction

The abscopal effect is a phenomenon seen when irradiation at a distinct anatomic site induces a systemic antitumor response throughout the body. It was first described using cell lines and was known as the “bystander effect.” Researchers found that in addition to direct cellular damage *via* reactive oxygen and nitrogen species induced by radiation therapy (RT), irradiated cells could also induce changes in distant non-irradiated cells through cell signaling molecules (1). Initial studies demonstrated that cell culture media taken from irradiated cultures could be transferred to non-irradiated cultures and induce DNA damage (1). Similarly, tumor cells can elicit cellular and DNA changes within normal cells when media used to grow tumor cells is transferred to normal cultures. This conditioned media demonstrates increased levels of many cytokines including transforming growth factor beta (TGFß) and C-C Motif Chemokine Ligand 2 (CCL2) (2, 3). In addition, *in vivo* experiments using both C57BL/6 wild-type and CCL2-knockout mice subjected to ionizing radiation identified six differentially expressed genes implicated in the abscopal effect in tissue outside the field of radiation. These include TGFß (4) and CCL2 (5), as well as tumor protein P53 (TP53) (6), tumor necrosis factor (TNF) (7), C-C Motif Chemokine Ligand 22 (CCL22) (8), and the proto-oncogene, MDM2 (9).

CCL2 is particularly important, as it is involved in the propagation of the immune effects associated with abscopal activity. Specifically, CCL2 is a member of the monocyte chemoattractant protein family and not only serves an important role in the recruitment of monocytes, but also has been shown to recruit T cells, B cells, NK cells, macrophages, and dendritic cells (10–14). It is induced by multiple pro-inflammatory molecules (15–18) and by reactive oxygen and nitrogen species generated by RT supporting the idea that it contributes significantly to the immune response associated with the abscopal effect (19).

While the precise mechanism for the abscopal effect is complex and continues to be elucidated, current evidence supports that it is primarily a T cell-mediated process. Ultimately, irreparable DNA damage in tumor cells induced by RT increases tumor immunogenicity by providing dendritic cells with tumor-specific antigens to present to, and activate, CD8^+^ T cells *via* major histocompatibility complex (MHC) class 1. Clinical case examples, which we summarize, have stimulated ongoing preclinical and clinical trials focusing on strategies to stimulate dendritic cell proliferation and T cell activation to more consistently induce an abscopal effect concurrent with RT.

In an effort to understand abscopal activity, we can look at the molecular mechanism behind RT-induced DNA damage and the immune response. Specifically, RT of tumor cells induces double-stranded DNA breaks and unique nucleotide adducts that leak into the cytosol and bind to the DNA sensor cyclic guanosine monophosphate-adenosine monophosphate (cGAMP) synthase (cGAS) protein resulting in an increase in intracellular cGAMP (20, 21). Increasing levels of cGAMP then bind to the stimulator of interferon genes (STING) protein leading to the production of type 1 interferons (IFN-1). (IFN-1 binds to IFNAR1/2 receptors that result in a signaling cascade that activates immune-stimulating genes that promote activation of dendritic cell populations (22).

However, in addition to the anti-tumor and immunogenic effects of the cGAS-STING pathway, this pathway has also been shown to induce pro-tumorigenic factors, such as IL-6 through activation of the NFkB pathway and PD-L1 through activation of the JAK-STAT pathway (23, 24). This is important in the context of induction of abscopal effect because IL-6 has been implicated in resistance to RT by suppressing oxidative stress, and efforts to pharmacologically block the production of IL-6, in addition to PD-L1, may help increase the abscopal effect (25). In addition to the canonical cGAS-STING pathway, an alternative STING pathway is also capable of sensing DNA damage independent of the cytosolic DNA receptor cGAS. Instead, this non-canonical STING pathway utilizes a protein complex consisting of a DNA binding protein called Interferon Gamma Inducible Protein 16 (IFI16), DNA damage response factors (ATM serine/threonine kinase [ATM] and Poly ADP-Ribose Polymerase 1 [PARP-1]), tumor suppressor protein p53 (TP53), and a E3 ubiquitin ligase called TNF Receptor Associated Factor 6 (TRAF6). This protein complex ultimately leads to the activation of the NFkB pathway resulting in expression of IFN-ß and thus its downstream targets (26). Each of these unique pathways demonstrates the complexity of RT-induced DNA damage and the diverse molecular mechanisms that play a role in both anti-cancer and pro-cancer response.

Importantly, as the dose of radiation increases, larger amounts of damaged DNA products precipitate in the cytosol activating TREX1, a cytosolic DNA exonuclease that functions to degrade and eliminate cytosolic DNA, precluding the activation of the cGAS-STING signaling cascade that is thought to trigger the abscopal effect. In an effort to fine-tune induction of abscopal effect, Vanpouille-Box et al. sought to determine an optimal radiation dose that maximized production of IFN-1 while minimizing TREX1 expression using a mammary carcinoma mouse model treated with RT and an antibody against anti-cytotoxic T-lymphocyte-associated protein 4 (CTLA-4). CTLA-4 is a cell-surface protein expressed by regulatory T cells to inhibit T cell functions by increasing the activation energy necessary for T cell activation (27). This is especially important in the context of cancer due to the weakly immunogenic self- and tumor-antigens. Ipilimumab is an anti-CTLA-4 antibody and was the first immune checkpoint inhibitor approved for treating cancer (28, 29). Their results showed that an irradiation scheme of 8 grey (Gy) x 3 coupled with an anti-CTLA-4 antibody did not result in TREX1 gene expression, while a single large dose (20 and 30 Gy) coupled with an anti-CTLA-4 antibody did (30). Activation of IFN-1 was preserved in both examples.

In addition to optimizing expression of both immune-stimulating and immune-suppressing genes, several studies have demonstrated the importance of an intact T cell response and a sufficient dendritic cell population to induce an abscopal response. For example, Stone et al. published one of the first preclinical experiments seeking to understand the abscopal effect using a syngeneic fibrosarcoma mouse model. They showed that the radiation dose necessary to reduce tumor size by 50% was significantly smaller in T cell-competent mice compared to T cell-depleted mice. In addition, they noticed that the likelihood of metastasis was lower in T cell-competent mice compared to their T cell-depleted counterparts (31). More recently, Demaria et al. utilized a murine model with both wild-type and *nu/nu* T cell-deficient BALB/C mice to compare (a) the effect of irradiation alone or irradiation supplemented with the dendritic cell stimulator, Fms Related Receptor Tyrosine Kinase 3 Ligand (Flt3-L), and (b) the effect of tumor immunogenicity using two cell lines (32). The two groups of mice were injected at two distinct anatomic sites with either the highly immunogenic 67NR BALB/C mouse-derived mammary carcinoma cell line or the low immunogenicity A20 BALB/C mouse-derived B-cell leukemia/lymphoma cell line creating a pseudo-primary site that would receive direct RT (2 Gy, single dose) and a secondary site that would not. Their results showed that not only were T cells necessary to induce a response at the secondary, non-irradiated site, the addition of Flt3-L significantly increased the tumor response at the non-irradiated site in the wild-type mice. In addition, the low immunogenicity A20 B cell leukemia/lymphoma cell line did not show a significant increase in response at the secondary site in both the irradiation alone and irradiation + Flt3-L groups demonstrating the importance of an immunogenic tumor in activating a T cell response.

The combination of RT with immune checkpoint inhibition (ICI)—pharmacologic agents has also resulted in a more potent tumor response than either treatment alone in preclinical studies examining head and neck cancer, metastatic melanoma, metastatic pancreatic cancer, and lung cancer (33–35). This has resulted in investigators examining ideal radiation dosing and fractionation schemes when coupled with ICI to induce abscopal responses. For example, Dewan et al. utilized a mouse model of bilateral mammary adenocarcinoma to identify an ideal radiation dose. In their study, they treated mice with either 3 fractions (8 Gy each) coupled with an anti-CTLA-4 monoclonal antibody (mAb), or a single dose (30 Gy) coupled with the anti-CTLA-4 mAb. Their results showed an abscopal response in the group treated with 8 Gy x 3 + anti-CTLA-4 mAb but did not see the same response in the group treated with a single dose of RT (30 Gy) coupled with the anti-CTLA-4 mAb (36).

## Clinical case reports of abscopal effect following irradiation without systemic treatment

Early evidence of abscopal activity has initially been portrayed in published case reports which are described below. Each demonstrate systemic clinical response following local site RT as consistent with abscopal activity.

A 57-year-old male was diagnosed with multiple lung nodules, vertebra metastases, and brain metastases (37). The results of pathological examination suggested adenocarcinoma of the lung. RT of 39 Gy in 13 fractions was administered to the ninth thoracic vertebra for destructive extension. However, all the lesions including the brain metastases spontaneously shrunk, thereby supporting abscopal activity as no systemic therapy had been administered. Two months after RT, complete regression to the lung and other non-irradiated thoracic vertebra was achieved. Whole-brain radiotherapy for a total dose of 36 Gy in 12 fractions was performed. Unfortunately,15 months after initial RT, the brain metastasis recurred.

A 61-year-old male with renal cell carcinoma and metastatic lesions to the brain, bone, spine, lung, and lymph nodes underwent stereotactic body radiation therapy (SBRT) to the brain metastases and external beam radiation therapy (EBRT) to the metastatic lesions in his bone and spine (38). 1 month later, lesions that were not subjected to radiotherapy showed regression as evidenced by CT scan. In addition, follow-up CT scans taken 2 months later and 3 months later demonstrated continued response of these untreated lung lesions suggesting a possible abscopal response. Unfortunately, this patient went on to develop new brain metastases requiring additional stereotactic radiosurgery.

A 66-year-old female with clear cell renal cell carcinoma was treated with a nephrectomy (39). Ten years later, the patient had a metastatic lesion of the renal cell cancer in the neck, and was treatment with pazopanib, but then terminated due to intolerability. CT scans of the thorax and the neck showed progression in the neck, portacaval lymph node, hypochondrium subcutaneous node, and new and progressive lung metastases. Palliative RT was given to the neck, but the patient did not resume systemic therapy. Eleven months after (radiation therapy) XRT, the patient had complete regression of the lung metastases, the subcutaneous abdominal node remained, but growth of the portacaval lymph node persisted. 17 months after XRT, the patient’s stable disease remained, demonstrating abscopal activity.

A 93-year-old female with melanoma on the fifth metatarsal (Breslow depth: 2.8 millimeters) underwent therapeutic amputation. Despite amputation, the patient demonstrated disease progression 13 months later. He presented with a 4-centimeter painful inguinal lymph node mass along with 5 cutaneous nodules located on the anterolateral leg below the knee. These cutaneous nodules were hard to palpation and macroscopically consistent with metastasis. The patient underwent palliative RT to the inguinal lymph node for pain management. Interestingly, the patient also demonstrated near complete resolution of the non-irradiated cutaneous lesions one month after RT. The patient was lost to follow-up 14 months later due to relocation to a new city, but demonstrated stable disease throughout that period (40).

A 75-year-old male with a history of stage IV colorectal cancer with liver metastasis (November 2007) status post anterior resection with partial hepatectomy and rectal cancer (January 2010) status post anterior resection presented with abdominal pain in November 2010. Abdominal imaging showed two masses: a 35-millimeter mass located on the left side of the abdomen and a 15-millimeter mass invading the right common iliac artery. The left-sided mass was irradiated using carbon-ion radiation therapy (CIRT) with a regimen of 73.6 Gy x 16 fractions over a 28-day period in January 2011. The mass invading the right common iliac artery was not irradiated due to its proximity to the small intestine. Interestingly, a PET-CT scan performed 1 month after therapy showed significant reduction in tumor size in both the irradiated and non-irradiated tumors as evidenced by decreased fludeoxyglucose accumulation. Unfortunately, the patient died 46 months after CIRT due to myelodysplastic syndrome with no evidence of progression of the two tumors as evidenced by annual PET-CT scans. Taken together, it is likely that this patient had a durable abscopal response to CIRT (41).

An 85-year-old male with a history of recurrent colon cancer in the ascending colon presented with back pain in February 2009 after a 10-month stable period post hemicolectomy (April 2008). CT imaging revealed a 45-millimeter para-aortic tumor along with two 10-millimeter tumors in the mediastinum and right clavicle. The patient was not eligible for chemotherapy due to comorbidities, so the decision was made to perform CIRT (Gy x 12 fractions over 21 days) on the para-aortic mass as part of an ongoing clinical trial. Following completion of therapy, there was a significant reduction in size of the irradiated para-aortic tumor as well as the non-irradiated mediastinal and subclavian tumors as evidenced by both CT and PET-CT imaging. The patient received no additional therapy, which suggests the patient had an abscopal response to CIRT. The patient’s condition has remained stable for 92 months at the time of publication with no change in tumor size (41).

A 63-year-old male presented with a 10.5 cm x 9 cm x 11 cm hepatocellular carcinoma (HCC) with 3 daughter nodules <1 centimeter each. He underwent an extended right lobectomy and was stable for 18 months until metastatic nodules were found in the right lower lobe of the lung and left mediastinal lymph node as evidenced by CT scan. This was confirmed to be HCC metastatic disease due to an alpha-fetoprotein (AFP) of 4,869 ng/mL and a protein induced by vitamin K absence or antagonists II (PIVKA-II) >20,000 mAU/mL. Trans-catheter arterial embolization of the mediastinal tumor was attempted but ultimately aborted due to risk of spinal artery embolism. The decision was made to perform palliative external beam radiation therapy (2.25 Gy x 27 fractions) on the mediastinal node. Following RT, both the mediastinal lymph node and the right lower lobe lung tumor demonstrated significant response as evidenced by CT scan along with a decrease in AFP from 4,869 ng/mL to 23 ng/mL and a decrease in PIVKA-II from >20,000 mAU/mL to 13 mAU/mL. His disease remained stable for 4 years until a 3.5 cm lymph node was found near the left gastric artery. He was treated with stereotactic body radiotherapy and showed no additional disease after 6 months (42).

A 76-year-old female was diagnosed with pulmonary adenocarcinoma (cT1bN0M0) in November 2015, and subsequently underwent a right upper lobectomy with confirmation of pathological pT1bN2M0, stage IIIA disease. The patient did not receive adjuvant chemotherapy. In February 2018, multiple new mediastinal and right hilar lymph node metastases were identified. A total dose of 60.0 Gy of RT was given over 6 weeks to selected lesions. The target area included multiple mediastinal, and several (but not all) right hilar lymph nodes. Twelve weeks after completion of RT, a chest CT scan showed complete disappearance of the treated and untreated pulmonary metastases. Another follow up CT scan was completed (6 months after completion of RT) showing no reappearance of multiple metastatic pulmonary nodules both non irradiated and irradiated pulmonary nodules supportive of abscopal effect (43).

An 81-year-old female was diagnosed with a pT2a, pN0 (0/5), cM0, UICC stage IB squamous cell carcinoma of the left upper lung lobe (44). She underwent a lobectomy with lymphadenectomy, and subsequently had no relapse for 5 years during follow up. Thirteen years later, recurrence was confirmed *via* biopsy, chest CT showed a mass in the left lung, negative for brain metastasis on MRI, and PET showing left sided pleural carcinomatosis, left sided periclavicular lymph node metastases, and bone metastases in the 12^th^ thoracic and 4^th^ lumbar vertebra. The patient declined systemic treatment. She thus underwent palliative radiotherapy to the symptomatic pulmonary tumor. Four weeks after RT completion, restaging was performed showing a partial remission of the tumor, the nodal metastases and the previously untreated vertebral lesions. During follow-up, further decrease in tumor size and complete metabolic remission of the bone, pleural and lymph node metastases was seen. 25 months after radiotherapy, the patient still had evidence of stable disease, but remained free of disease symptoms.

In June 2018, a 69-year-old male was diagnosed with squamous cell carcinoma of the right lower lobe with involvement of mediastinal nodes (45). The patient was initially treated with vinorelbine and cisplatin however, after four cycles, his symptoms worsened, and chest CT scan confirmed progressive disease. Hence, the chemotherapy regimen was shifted to paclitaxel, but the primary lung lesion was still not controlled, and he showed disease progression in the chest, and as well as a bone scan that showed a new lesion in the right tibia, indicating the occurrence of bone metastases. After initial response, this patient showed progression on the PD-1 inhibitor, camrelizumab, and the tyrosine kinase inhibitor that selectively inhibits VEGFR2, apatinib, and went on to receive palliative CT-guided microwave ablation to the primary lung tumor. One month later, chest CT scan showed the right lower lobe mass and mediastinal lymph nodes were also reduced, indicating an abscopal effect following local ablation.

These cases highlight systemic abscopal effect related to localized RT. The effect involved a broad range of cancer patients that include a robust age range up to 93 years old.

## Case reports of abscopal effect with irradiation and enhancing immune modulation

Evidence of abscopal activity related to systemic immune induction of RT may be enhanced with combination immune modulatory therapy. The following case reports support evidence of abscopal activity with combined RT in a setting of failed systemic response prior to ongoing immunotherapy followed by immune response with same immune therapy (abscopal effect) after local RT.

A 54-year-old male patient presented with a stageT4N0M1b disease. He had a pulmonary large cell neuroendocrine carcinoma of the right upper lobe, associated with bilateral adrenal metastases and a PD-L1 tumor proportion score of 20% (46). After four cycles of chemotherapy (pemetrexed, cisplatin) and the VEGF inhibitor, bevacizumab, CT scans revealed disease progression in the right upper lobe as well as in both adrenal glands. Second-line therapy with nivolumab, a PD-1 inhibitor, was started, but increasingly symptomatic spinal cord compression, due to tumor invasion occurred. Hemilaminectomy of the third thoracic vertebra combined with resection of the epidural tumor mass was thus performed. Postoperative radiotherapy (30 Gy) was applied targeting the involved thoracic vertebrae. Nivolumab continued, CT scans 4 months after the first radiotherapy showed partial regression of the lung tumor and adrenal metastases. The patient showed disease progression 10 months after radiotherapy but is still alive, supportive of abscopal effect, 25 months after the initial diagnosis.

A 64-year-old male patient presented with T2N3M1c disease which included an adenocarcinoma of the left upper lobe, mediastinal contralateral lymph nodes and distant metastases (brain and ocular). The patient’s PD-L1 results are currently blinded due to the requirements of a clinical trial (Impower130 trial: ClinicalTrials.gov identifier NCT02367781). The radiological images after 5 months of treatment (four cycles of nab-paclitaxel/carboplatin with atezolizumab, a PD-L1 inhibitor, followed by four cycles of atezolizumab alone) showed an excellent response of the ocular metastasis, but progression of the brain metastasis. The thoracic tumor manifestations showed partial remission after four cycles of combined chemotherapy and immunotherapy with no further shrinkage after the four additional cycles of atezolizumab monotherapy. Whole-brain radiotherapy (WBRT) was performed and atezolizumab was continued. Radiological follow-up 4 months after WBRT showed a partial response in the brain (complete response [CR] of ocular disease and remission of brain disease) as well as complete remission of lung and mediastinal tumor masses, supportive of potential abscopal effect. The patient is still alive with radiologically nearly complete remission 28 months after the initial diagnosis of metastatic lung cancer (46).

A 70-year-old male patient presented with a T3N2M1a disease involving central adenocarcinoma of the middle lung lobe, associated with positive mediastinal lymph nodes and a malignant ipsilateral pleural effusion. There were no EGFR or ALK mutations and the PD-L1 tumor proportion score was 70%. First-line therapy with pembrolizumab, a PD-1 inhibitor, was started, leading to a partial response. After a year of treatment, pulmonary and pleural disease progression occurred, and a clinically symptomatic brain metastasis associated with perimetastatic cerebral edema appeared. Pembrolizumab was continued and WBRT added (30 Gy in ten fractions). Radiography of the thorax after radiotherapy showed partial regression of the lung tumor and pleural effusion, supportive of abscopal activity. The patient is still alive 19 months after the initial diagnosis (46).

A 65-year-old female presented with mucosal melanoma in June 2015 with no evidence of metastatic disease as evidenced by CT scan of the neck and MRI with gadolinium. The decision was made to perform a right partial maxillectomy to remove the lesion followed by targeted intensity modulated radiotherapy (IMRT). Unfortunately, the patient relapsed 9 months later, and evidence of disease progression was found in the neck and lungs. The patient was then enrolled in a trial comparing epacadostat + pembrolizumab or placebo + pembrolizumab, which initially showed tumor response, but ultimately resulted in disease progression. The treatment was stopped, and the patient started a palliative course of IMRT to the neck due to increased symptoms. Interestingly, both the neck lesion and the pulmonary lesions responded to IMRT based on CT scans before and after IMRT suggesting an abscopal response (47).

A 67-year-old female presented with metastatic pancreatic uncinate carcinoma to the right liver lobe in August 2015 with a CA 19-9 of 1,814 U/mL. The patient was initially treated with single-agent gemcitabine, but this was discontinued due to poor response and worsening abdominal pain. The patient was then switched to albumin-bound paclitaxel, which demonstrated partial response based on Response Evaluation Criteria in Solid Tumors (RECIST 1.0), but ultimately demonstrated disease progression with additional metastasis to the right pleura and worsening side effects. The patient was then switched to Apatinib, but this was quickly discontinued due to severe gastrointestinal distress. The decision was made to initiate palliative radiotherapy (45 Gy x 15 over 3 weeks) coupled with GM-CSF to the primary pancreatic tumor due to abdominal pain and jaundice requiring percutaneous transhepatic-cholangial drainage. 1 month later, the patient demonstrated significant response to the primary tumor as evidenced by CT scan, but also demonstrated significant abscopal response to the metastatic sites in the liver and pleura that were outside the cone of radiation (48).

A 33-year-old female presented with a mole on her upper back concerning for melanoma in April 2004. Biopsy of the lesion revealed melanoma with a Breslow thickness of 1.53 millimeters. The decision was made to perform a wide local excision of the malignant lesion along with sentinel lymph node biopsy. The patient remained disease free until 2008 when a PET-CT revealed a 2-centimeter pulmonary nodule suggestive of metastatic disease that was confirmed *via* CT percutaneous biopsy. She was treated with cisplatin, vinblastine, and temozolomide (CVT) chemotherapy due to lack of a targetable mutation (e.g., BRAF) followed by surgical resection. The patient demonstrated stable disease until surveillance CT scan showed a metastatic paraspinal mass along with hilar lymphadenopathy in August 2009. The decision was made to initiate 4 doses of ipilimumab, a CTLA-4 inhibitor, (10 mg/kg) every 3 weeks which resulted in an initial slight enlargement of the paraspinal mass but effectively stabilized her disease for 14 months. Unfortunately, the patient demonstrated continued enlargement of the paraspinal mass with additional evidence of metastatic splenic lesions. The patient was experiencing significant back pain due to mass effect from the paraspinal mass, so the decision was made to initiate palliative RT to the paraspinal mass (950 Gy x 3 fractions over 7 days). Ten months after therapy, there was evidence of abscopal effect as evidenced by CT-scan demonstrating significant reduction in size of both the treated paraspinal mass and the splenic lesions that were outside the cone of radiation (49).

A 71-year-old male was diagnosed with stage IV lung adenocarcinoma, and began treatment with atezolizumab (50). After 19 months of atezolizumab, there was a complete response to the primary lung tumor. A brain metastasis then developed two years later, which was treated with gamma knife radiotherapy. However, after radiation, the patient’s lung disease recurred. Two months later, the lesions in the lung had shrunk, indicating that the prior changes in the lung may have been pseudo-progression as abscopal effect was later demonstrated.

Combination irradiation with immunotherapy may be associated with more frequent abscopal effect as suggested by preclinical testing and preliminary clinical results (31–36, 51). In summary (see **Tables 1**, **2**), these 17 case reports provide evidence of abscopal effect and support combination with immunotherapy is well tolerated and may enhance abscopal activity related to RT.

**Table 1:** Abscopal case reports following irradiation without systemic treatment.

Patient	Disease	Sites of involvement	Treatment	Response	Reference
57-year-old male	Unknown primary	Lung nodules, vertebra, and brain metastases	Radiation (XRT) to 9^th^ vertebra	All lesions	(37)
61-year-old male	Renal Cell carcinoma	Bone, spine, brain, lung lymphadenopathy mets	XRT to brain, spine, and bone	Regression of untreated lung metastases and lymphadenopathy	(38)
66-year-old female	Renal Cell carcinoma	Neck, lung and portacaval node	XRT to the neck	Regression of irradiated neck mass and non-irradiated lung metastases	(39)
93-year-old female	Melanoma (toe)	Thigh and inguinal node	Surgery, RT to inguinal region	Regression of thigh lesions	(40)
75-year-old male	Colorectal cancer	Liver mets	Resection and RT	Reduction in both the treated and untreated liver masses	(41)
85-year-old male	Colorectal Cancer	Nodes (near abdominal aorta, mediastinal, and subclavian)	Resection and XRT to aortic lymph node	Untreated subclavian node shrank, and mediastinal node remained stable.	(41)
63-year-old male	Hepatocellular Carcinoma	Lung and mediastinal nodes	XRT to mediastinal lymph node	Mediastinal lymph node and untreated lung mass	(42)
76-year-old female	NSCLC	Mediastinal and hilar lymph nodes, lung disease	XRT only, to mediastinal and hilar lymph nodes.	Complete response to multiple untreated lung lesions	(43)
81-year-old female	NSCLC	Lung, pleura, periclavicular node, and vertebra	XRT only	Complete remission of untreated vertebral lesions and periclavicular node	(44)

**Table 2:** Abscopal case reports of irradiation and systemic treatment following systemic treatment failure.

Patient	Disease	Sites of involvement	Treatment	Response	Reference
69-year-old male	NSCLC	Lung, mediastinal and other nodes	Vinorelbine/cisplatin, paclitaxel, camrelizumab/apatinib, CT-guided microwave ablation of lung lesion, following systemic treatment failure	After progression on systemic treatment and then XTR, right lower lobe mass and mediastinal lymph nodes reduced	(45)
54-year-old male	Neuroendocrine	Lung, adrenal glands, para-spinal cord	After progression with systemic therapy, Pemetrexed/cisplatin/bevacizumab, nivolumab, XRT to para spinal vertebrae	Lung tumor and adrenal metastases underwent regression	(46)
64-year-old male	NSCLC	Mediastinal contralateral lymph nodes and distant metastases (brain and ocular)	Nab-paclitaxel/carboplatin/atezolizumab, atezolizumab alone, after progression of all lesions, WBRT (whole brain radiation therapy)	PR to brain, CR to lung and mediastinal masses was seen after WBRT	(46)
70-year-old male	NSCLC	Mediastinal lymph nodes and malignant pleural effusion, PD-L1 70%	Failed Pembrolizumab, then WBRT added	Partial regression of the lung tumor and pleural effusion after WBRT	(46)
65-year-old female	Melanoma (mucosal)	Neck and pulmonary mets	Failed Pembrolizumab, then given XRT to neck	Tumor regression of the pulmonary metastases after XRT to the neck	(47)
67-year-old female	Pancreas Cancer	Liver and right pleura	Failed Gemcitabine, paclitaxel, apatinib, then given palliative XRT (to pancreas)/GM-CSF	XRT to pancreatic tumor, non-irradiated systemic metastases significantly decreased	(48)
33-year-old female	Melanoma (cutaneous)	Pulmonary nodule, paraspinal mass, hilar node, splenic lesion	Failed Cisplatin/vinblastine/temozolomide (CVT), ipilimumab, XRT to paraspinal mass	Regression to non-irradiated hilar lymphadenopathy and splenic lesions	(49)
71-year-old male	NSCLC	Brain mets, mediastinal lymph nodes lung disease	Failed nedaplatin/paclitaxel, and Atezolizumab, then given Atezolizumab/brain XRT	Primary lung lesion and hydrothorax decrease after systemic treatment failure and brain XRT	(50)

## Current studies evaluating abscopal effect

The clinical trial landscape for abscopal effect and development of clinical trials is increasing. There are several prospective clinical trials investigating the abscopal effect (**Table 3**). However, consistent results regarding occurrence of abscopal effect and level of benefit are highly variable. Moreover, trials exploring expansion of abscopal effect looking at various irradiation doses, schedules and immune modulating combination therapy still only provide relatively low occurrence rate of abscopal activity. In general, though, when abscopal effect is observed compared to those receiving the same regimen without abscopal effect clinical benefits with respect to response, progression-free survival (PFS) and overall survival (OS) is observed (52–54). In one early retrospective study involving melanoma patients treated with ipilimumab followed by RT, 52% showed evidence of abscopal activity and those who did had significantly improved OS (54). Another retrospective study involving melanoma showed similar results (55). Moreover, in a third small trial of 10 prostate cancer patients improved durable disease control was observed with combined ipilimumab and irradiation (56). Although in a larger later Phase 3 trial of advanced prostate cancer undergoing irradiation and ipilimumab vs. irradiation alone OS was not different (57).

**Table 3:** Select ongoing clinical trials involving radiotherapy and checkpoint inhibitors to achieve abscopal effect.

Cancer type	Irradiation scheme/combination	Clinical trial number
NSCLC	30-50 Grays in 5 fractions Bevacizumab toripalimab	NCT04238169
NSCLC	20 Gray nivolumab	NCT03480334
NSCLC	20 x 2 Gray (daily for 4 weeks) 5 x 5 Gray (daily over 1 week) 3 x 8 Gray (every other day over 1 week) Durvalumab	NCT04245514
Metastatic Cancer	1 dose of SBRT Durvalumab and trememlimumab	NCT03212469
Hepatocellular	4 fractions over 8-15 days Pemrolizumab	NCT03316872

Similarly, several combination PD-1/PD-L1 checkpoint inhibitor treatments with RT have also demonstrated evidence of abscopal activity. KEYNOTE-001 trial demonstrated improved PFS and OS in NSCLC patients who received prior RT and pembrolizumab compared to pembrolizumab alone although actual abscopal events were not well defined (58). Another retrospective study looking at PD-1 inhibitors involving melanoma patients, some receiving RT, showed significant improvement in response rate but no improvement in PFS and OS with combination checkpoint inhibitors/RT. However, only one patient of 59 demonstrated abscopal activity (59). Not all clinical results have reproduced the same result. For example, a Phase I clinical trial examining the ideal radiation dose in patients with metastatic NSCLC or melanoma on pembrolizumab showed abscopal responses in patients treated with either 24 Gy x 3 fractionation scheme or a single 17 Gy fraction (NCT02303990) (60). This suggests that the abscopal response to irradiation is multi-factorial and radiation fractionation regimens may not be universal.

Interestingly, GM-CSF combination RT involving a general group of 41 solid tumor patients showed a high fraction (over 25%) of patients with abscopal activity (breast cancer, NSCLC, thymic cancer) when combined with localized irradiation (61). In addition, Formenti et al. performed a proof-of-principle trial where they supplemented RT with subcutaneous GM-CSF, a cytokine that promotes dendritic cell differentiation and expansion, in patients with metastatic tumors including breast cancer, bladder cancer, and eccrine cancer (51, 62). Their results showed that 30% of patients who received RT supplemented with GM-CSF over the course of 2 weeks had an abscopal response as evidenced by PET/CT. Also, breast cancer patients receiving high dose vs. low dose TGFβ blockade (fresolimumab) along with RT had significantly prolonged OS (63).

## Consideration GM-CSF expression/TGFβ knockdown to induce abscopal effect

There continues to be strong evidence that radiation is able to activate the immune system although the mechanism for this has not been fully elucidated (62). RT has also been shown to promote the development of an immunosuppressive tumor microenvironment, specifically by upregulation of PD-L1 (64, 65). Therefore, it has been hypothesized that radiation coupled with immunotherapy would elicit an abscopal effect. However, results have been limited with studies evaluating the dose of radiation and sequencing of combination immunotherapy (66). The abscopal effect has largely been observed in highly immunogenic tumors including melanoma, renal cell, and hepatocellular carcinoma. The tumor microenvironment in these “hot” tumor types are characterized by T cell infiltration and expression of proinflammatory cytokines (67).

In addition to combination with checkpoint inhibitor therapy, autologous tumor cellular immunotherapy may also be considered as a clinical testing direction. Vigil is a triple function immune therapy constructed from patient tumor cells. Vigil mechanism involves the introduction of bifunctional short-hairpin RNA to knockdown furin in the autologous tumor cells. Furin knockdown results in decreased cleavage of TGFβ into TGFβ1 and TGFβ2 (68). TGFβ is an immune suppressive cytokine associated with poor prognosis and therapeutic resistance in many solid tumors (69–71). Vigil plasmid also encodes for human GM-CSF which is also an immune stimulatory cytokine that increases tumor antigen presentation by dendritic cells (72). Moreover, Vigil provides personalized, clonal cancer specific neoantigens to enable the immune system to recognize tumor cells and mount an effective, targeted T-cell mediated response. Vigil has demonstrated improved clinical outcomes which correlated with IFNγ-ELISPOT positivity (73, 74). IFNγ is known to activate a multitude of immune cells, including effector T cells. Vigil has shown clinical benefit in advanced solid tumor patients with overall survival correlation with TIS^HIGH^ vs. TIS^LOW^ (one year OS 75% vs. 25%, p=0.03795) and elevated MHC-II expression (p=0.038). In recurrent ovarian cancer patients, the OS rate was observed to be 58% compared to historical rate of <20% with standard of care. In a Phase IIb double-blind, randomized, placebo-controlled trial in frontline ovarian cancer maintenance, Vigil patients demonstrated a trend towards benefit (11.5 months vs. 8.4 months for placebo, p=0.078). The result for the secondary endpoint of recurrence-free survival (RFS) for the BRCA-wt subpopulation however, was statistically significant, demonstrating benefit in RFS from procurement (time of initial debulking surgery) and randomization (time of initial Vigil administration; 18.3 months vs. 14.8 months, HR=0.478, p=0.02; 11.5 months vs. 8.0 months, HR=0.514, p=0.02 respectively) and OS from procurement and randomization (not reached vs. 48.3 months, HR=0.490, p=0.047; not reached vs. 41.4 months, HR=0.493, p=0.049 respectively). Based on a *post hoc* exploratory analysis in the BRCA-wt, HRP subpopulation, RFS and OS were increased in a statistically significant fashion relative to the control arm, demonstrating a benefit with Vigil in RFS from procurement and randomization (18 months vs. 12 months, HR=0.363, p=0.005; 10.6 months vs. 5.7 months, HR=0.386, p=0.007 respectively) and OS from procurement and randomization (not reached vs. 37.3 months, HR=0.340, p=0.018; not reached vs. 26.9 months, HR=0.342, p=0.019 respectively). Long term follow up analysis also revealed that 83% of Vigil treated patients were still alive three years after their initial debulking surgery versus 40% who received placebo (p=0.0006). Clinical testing of Vigil with RT to induce and augment the abscopal effect is under consideration.

## Conclusion

Clearly sufficient preclinical and clinical evidence exists which support benefit to patients who incur abscopal effect while undergoing RT. There does not appear to be any concerning toxic effect related to abscopal activity. Benefit associated with response, PFS, duration of PFS and OS has been observed. However, results are inconsistent and hard to predict. Biomarkers indicative of abscopal development are not known. Combination of RT with immune modulatory therapy appear to suggest enhancement in abscopal activity but results are variable. Further research towards enhancement in abscopal activity is warranted. Consideration in modulation of GM-CSF expression and TGFβ knockdown is justified.

## Data availability statement

The original contributions presented in the study are included in the article. Further inquiries can be directed to the corresponding author.

## Author contributions

DC, SA, LS, and AW contributed to writing and editing the manuscript. JN was responsible for manuscript conception, supervision and writing and editing. All authors contributed to the article and approved the submitted version.

## Conflict of interest

Authors SA, LS, AW, and JN were employed by Gradalis, Inc.

The remaining author declares that the research was conducted in the absence of any commercial or financial relationships that could be construed as a potential conflict of interest.

## Publisher’s note

All claims expressed in this article are solely those of the authors and do not necessarily represent those of their affiliated organizations, or those of the publisher, the editors and the reviewers. Any product that may be evaluated in this article, or claim that may be made by its manufacturer, is not guaranteed or endorsed by the publisher.

## Abbreviations

AFP, alpha-fetoprotein; ATM, ATM serine/threonine kinase; CCL2, C-C Motif Chemokine Ligand 2; CCL22, C-C Motif Chemokine Ligand 22; cGAMP, cyclic guanosine monophosphate-adenosine monophosphate synthase; cGAS, cGAMP synthase; CIRT, carbon-ion radiation therapy; CR, complete response; EBRT, external beam radiation therapy; Flt3-L, Fms Related Receptor Tyrosine Kinase 3 Ligand; Gy, grey; HCC, hepatocellular carcinoma; ICI, immune checkpoint inhibitor; IFI16, interferon gamma inducible protein 16; IMRT, intensity modulated radiotherapy; MDM2, MDM2 proto-oncogene; MHC, major histocompatibility complex; OS, overall survival; PARP-1, poly ADP-ribose polymerase 1; PFS, progression-free survival; PIVKA-II, protein induced by vitamin K absence or antagonists II; RFS, recurrence-free survival; SBRT, stereotactic body radiation therapy; STING, stimulator of interferon genes; TGFß, transforming growth factor beta; TNF, tumor necrosis factor; TRAF6, TNF receptor associated factor 6; TP53, tumor protein P53; WBRT, whole-brain radiotherapy; XRT, radiation therapy.
